# Quantitative interocular suppression in children with intermittent exotropia

**DOI:** 10.3389/fnins.2023.1204061

**Published:** 2023-08-03

**Authors:** Hui Chen, Xiaohui Jiang, Weijie Liu, Jiawei Zhou, Jie Chen, Qianqian Sun, Lin Liang, Jiangtao Lou, Xinping Yu, Jia Qu

**Affiliations:** ^1^National Clinical Research Center for Ocular Diseases, Eye Hospital, Wenzhou Medical University, Wenzhou, China; ^2^State Key Laboratory of Ophthalmology, Optometry and Vision Science, Eye Hospital, Wenzhou Medical University, Wenzhou, China; ^3^State Key Laboratory of Ophthalmology, Zhongshan Ophthalmic Center, Sun Yat-sen University, Guangzhou, China

**Keywords:** intermittent exotropia, interocular suppression, quantitative detection, interocular luminance differences, binocular imbalance

## Abstract

**Purpose:**

We have demonstrated that the depth of unbalanced interocular suppression can be quantified by balancing the interocular luminance differences required when both eyes are viewing simultaneously. In this study, we aimed to investigate the applicability of this method in children with intermittent exotropia (IXT), offering a quantitative assessment of interocular suppression in individuals with binocular imbalance. Additionally, we evaluated its association with the clinical characteristics of IXT.

**Methods:**

Interocular suppression in IXT was quantitatively measured using a polarizer and neutral-density (ND) filters. The density of the ND filter was adjusted incrementally from 0.3ND to 3ND, with a step size of 0.3ND (a total of 10 levels). Our prospective study involved 46 patients with IXT (mean age: 10.12 ± 4.89 years; mean ± SD) and 24 normal observers (mean age: 7.88 ± 1.83 years).

**Results:**

The suppression test exhibited good test–retest reliability, supported by statistical analysis. We observed more pronounced interocular suppression in individuals with IXT compared to controls. Notably, the magnitude of suppression during distant and near viewing significantly differed in IXT (1.55 ± 0.93 vs. 0.57 ± 0.64; *Z* = 4.764, *p* < 0.001). Furthermore, we identified a positive correlation between interocular suppression and data obtained from the Worth-4-Dot test. Additionally, interocular suppression showed a significant association with distance control scores.

**Conclusion:**

Our novel test offers a convenient and reliable means to quantify interocular suppression in patients with IXT. The quantitative assessment of interocular suppression provides a sensitive tool to evaluate the clinical characteristics of IXT.

## 1. Introduction

Intermittent exotropia (IXT) is the most prevalent form of exotropia (Govindan et al., 2005), affecting approximately 1% of children in the United States and up to 3.5% in Asia (Fu et al., 2014). Currently, the evaluation of IXT patients primarily relies on the assessment of their deviation angle, stereoacuity and control. However, these three clinical characteristics of IXT individuals often exhibit inconsistencies (Stathacopoulos et al., 1993), leading to challenges in clinical judgment and diagnosis.

Previous studies have indicated the involvement of interocular suppression in IXT, both in cases with deviation (Pratt-Johnson and Wee, 1969; Pritchard and Flynn, 1981; Melek et al., 1992; Cooper et al., 2000) and even during orthopia (Serrano-Pedraza et al., 2011). Rosenbaum et al. reported that the development of suppression in IXT may occur prior to the loss of distance stereoacuity, suggesting that evaluating interocular suppression could aid in the early detection and identification of IXT (Sloper, 2000). Distance suppression has also been investigated as a predictive factor for the severity of intermittent exotropia and treatment outcome (Yoo et al., 2019). Furthermore, patching therapy has shown promise in improving exotropia control and reducing the degree of exotropia in children with IXT (Cotter et al., 2014; Kushner, 2019; Gökgöz Özişik et al., 2022). Taken together, these findings suggest that mitigating interocular suppression could be a practical approach for treating IXT.

There is a need for a quantitative method to evaluate interocular suppression in children with IXT. Existing methods include qualitative approaches such as the Worth 4-dot test and the use of Bagolini glasses, as well as quantitative methods like electrophysiological (Brown et al., 1999; Zheng et al., 2019) and neuroimaging (Lygo et al., 2021) techniques, and various established psychophysical paradigms such as global motion coherence thresholds, direction coherence, interocular phase combinations (Narasimhan et al., 2012; Ding et al., 2013; Wang et al., 2019; Zheng et al., 2019; Gong et al., 2020; Lygo et al., 2021), and the dichoptic optokinetic nystagmus test (Cai et al., 2021). However, these assessments often require extensive cooperation from patients and can be particularly demanding for young children. Moreover, a comprehensive evaluation of the association between interocular suppression and fusion, stereo function, fusion control, and deviation in IXT has not been conduct.

Previous studies (Zhou et al., 2013a,b) have shown that when monocular luminance is adjusted, the perceptual dominance of both eyes changes. This phenomenon is explained by a brightness-adjusted contrast gain control model (Zhou et al., 2013b), which suggests that lower input luminance in one eye reduces the contrast gain of that eye, thereby transferring perceptual dominance to the other eye. These results suggest that the depth of unbalanced interocular suppression can be quantified by balancing the interocular luminance differences required when both eyes are viewing simultaneously. In previous studies (Chen et al., 2019a,b), we developed a method, which has good repeatability and consistency in amblyopic children, to quantify interocular suppression. This method involves presenting black-and-white striped butterfly stimuli dichoptically through polarized glasses, and quantifying interocular suppression by the interocular luminance difference required when both eyes are discriminating the stimuli. In this study, we intend to explore whether this method can be used to evaluate interocular suppression in children with IXT and the relationship between interocular suppression and the clinical features of IXT.

## 2. Materials and methods

This prospective study was approved by the Ethics Committee of the Eye Hospital of Wenzhou Medical University, and all procedures adhered to the principles in the Declaration of Helsinki.

### 2.1. Participants

A total of 46 patients with IXT (mean age: 10.12 ± 4.89 years, mean age ± SD; 23 females) and 24 age-matched normal subjects (mean age: 7.88 ± 1.83 years; 14 females) participated in the main study. An additional 20 IXT patients (mean age: 10.62 ± 2.20 years; 11 females) participated in two separate tests to assess the retest reliability of the suppression test. Clinical and demographic details of the 46 enrolled patients are provided in Table 1.

**Table 1:** Clinical details of the patients.

Characteristics	Data
Number of male participants	23 (50%)
Age, years	10.2 ± 4.9
Distance exodeviation, prism diopter	20.3 ± 8.8
Near exodeviation, prism diopter	23.6 ± 9.2
Control score at distance	1.8 ± 1.2
Control score at near	1.4 ± 1.2
Stereoacuity (Optec6500, arc sec)	450 ± 330
Stereoacuity (TNO, arc sec)	161 ± 138
Worth-4-Dot test at distance	0.8 ± 0.7
Worth-4-Dot test at near	0.4 ± 0.7
Spherical equivalent (OD/OS, diopter)	−1.79 ± 2.06/−1.98 ± 1.91

The inclusion criteria for participants with IXT in the current study were as follows: (1) diagnosis with the basic type of IXT; (2) no history of amblyopia in either eye; (3) absence of diplopia; (4) absence of any other eye diseases apart from IXT and refractive errors; and (5) no systemic diseases.

The following criteria were used for exclusion: (1) amblyopia (Snellen visual acuity chart ≥2 lines); (2) anisometropia (spherical or cylindrical difference ≥ 2D); (3) history of previous strabismus surgery or binocular vision training; (4) constant tropia (control score, 5) observed at both distance and near; (5) history of other ocular disease or surgeries; (6) presence of neurologic abnormalities, organic eye diseases, and/or developmental delays; and (7) uncooperative subjects.

All normal subjects had emmetropic eyes with uncorrected visual acuity ≤0.0 logMAR, thus they did not require optical correction during the test. Refractive errors in participants with IXT were corrected with glasses during the test.

### 2.2. Data collection

The clinical examinations encompassed the collection of gender and age information, as well as quantitative measurements of interocular suppression, evaluation of control, stereoacuity, Worth-4-Dot test, and angle of deviation. To minimize the potential impact of PACT and the stereopsis test on the results of interocular suppression, we specified the order of these clinical examinations as described above. The prism and alternate cover test (PACT) was employed to measure the deviation angle (PD) at both distance (6 m) and near (0.33 m) following refraction correction.

### 2.3. The quantitative interocular suppression test

**Figure 1:**
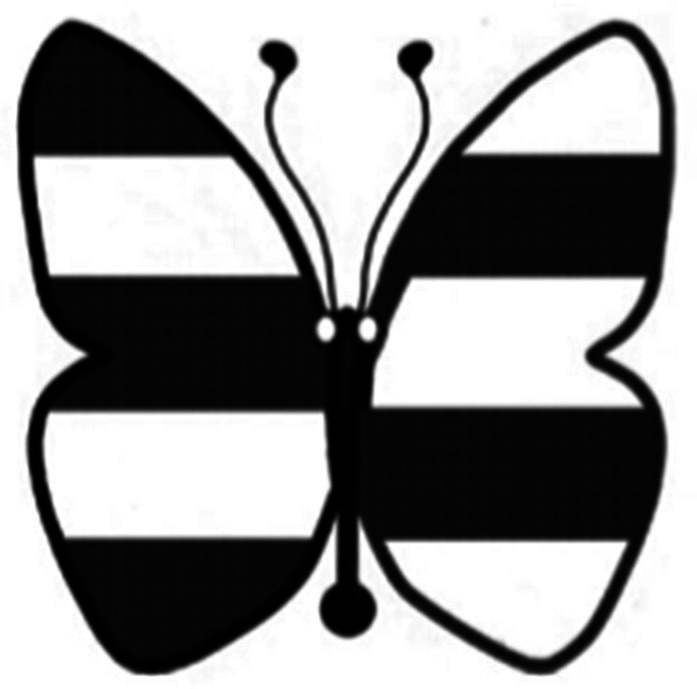
The visual stimuli of measuring interocular suppression test. It is a left–right symmetrical icon with a number of striped areas on the icon. The spacing and thickness of each area of the striped region are the same (Chen et al., 2019a). The left and right wings of the butterfly were presented to the left and right eyes of the subject wearing polarized glasses, respectively.

The measurement method used in this study has been previously reported (Chen et al., 2019a,b). A polarizing filter was used to present a black and white (i.e., full contrast) butterfly stimulus (Figure 1) to the observer, who was asked to report whether the left wing was as bright as the right wing. The density of the neutral-density (ND) filter was adjusted in increments of 0.3ND (10 levels) ranging from 0.3ND (transmittance 50%) to 3ND (transmittance 0.098%) until the observer perceived both wings as equally bright. The corresponding density of the ND filter (measured in units of ND) served as an index of interocular suppression. An interocular suppression index of 0 ND indicated balanced suppression, while a higher value indicated a greater imbalance in interocular suppression (Chen et al., 2019a). Additionally, the test–retest reliability of the measurement was assessed.

Interocular suppression was measured in a dimly lit room (15 lux) during both far distance (5 m) and near distance (0.33 m) viewing. The visual target size was 1.81 cm × 1.81 cm (3.14° × 3.14°) for near distance and 7.27 cm × 7.27 cm (0.8° × 0.8°) for far distance (equivalent to Snellen visual acuity of 20/200). The butterfly pattern stimulus was printed on transparent organic glass paper, with a backlight illuminance of 320 cd/m^2^ (Chen et al., 2019a).

Prior to testing, participants were provided with practice tests to ensure their understanding of the tests. Two measurements were taken for 20 patients with IXT within 1–2 weeks to assess the reliability of the test. The same experimenter completed the first and second tests, and did not view the results of the first test during the second test.

Suppression in IXT patients could shift depending on their fixation (Serrano-Pedraza et al., 2011; Wakayama et al., 2013; Adams et al., 2017; Ramachandran and Das, 2020). Therefore, when we performed the suppression test in 20 cases of IXT, we used an eye tracker (Tobii pro glasses 3: Tobii Technology, Inc., United States)^1^ to monitor whether the eye position remained the same across the two separate test sessions.

### 2.4. Evaluation of control

To assess the control of exotropia in each patient, a simplified control scale was employed, ranging from 0 to 5 for both distance (3 m) and near (0.33 m) evaluations (Mohney and Holmes, 2006). This simplified scale is consistent with the Evaluation of Control grading standard proposed by Mohney and Holmes (2006) and aligns with the conclusions of the Newcastle Control Score (Table 2).

**Table 2:** Control score description and instructions.

Control score	Control score description
5	Constant exotropia during a 30 s observation period (before dissociation)
4	Exotropia>50% of the time during a 30 s observation period (before dissociation)
3	Exotropia<50% of the time during a 30 s observation period (before dissociation)
2	No exotropia unless dissociated (10 s): recovery in > 5 s
1	No exotropia unless dissociated (10 s): recovery in 1–5 s
0	Pure phoria: <1 s recovery after 10 s dissociation

### 2.5. Instructions

During a 30-s observation period, the levels 5–3 are assessed. If exotropia is observed, testing is stopped and the control score is recorded as 5, 4, or 3, respectively, at that distance. If no exotropia is observed during the 30-s observation period, testing continues. The levels 2–0 are then assessed and graded, based on the worst of three consecutive 10-s periods of occlusion. First, an occluder is placed over the right eye for 10 s, and the time required for fusion is noted. Similarly, the left eye is occluded for 10 s, and the time to refusion is measured. A third occlusion trial is performed on the eye that required the longest time to refuse. The worst of the three 10-s trials is recorded, resulting in a control score of 2, 1, or 0, respectively, at that distance.

### 2.6. Stereo acuity test

Stereo vision at far distance was measured using the Optec 6500 vision tester (Stereo Optical Company, Chicago, IL). Near (0.33 m) stereoacuity was measured using the TNO stereo test (TNO 13, Lameris Ootech BV, Celsiusbaan 6B, 3439 NC, Nieuwegein, Netherlands). Stereoacuity was recorded as “800” if the patient could not pass the test at maximum disparity.

### 2.7. Worth-4-Dot test

The classic Worth-4-Dot test was conducted at both far distance (5 m) and near distance (0.33 m). In the test, participants were required to report the color and number of dots they saw under bright viewing conditions (220 lux). The brightness of the Worth-4-Dot display was set at 35 cd/m^2^. The Worth-4-Dot test was utilized to assess suppression, and for the quantification of the results, different people may use different ways, we were referring to previous study and took a similar approach to do such quantification (Li et al., 2011; Chen et al., 2019a): If a patient reported seeing four dots and the bottom dot was white, indicating binocular perceptual fusion, so 0 was used to indicate no suppression. When the color at the bottom alternated rapidly between red and green, this indicated that the two eyes were competing and also indicated no suppression, indicated by a 0 (i.e., no suppression). If a patient reported seeing four dots and the perceived color of the bottom dot was green or red, it indicated either left-eye dominance or right-eye dominance, so it was considered partially suppressed, then the index was assigned a value of 1 (i.e., partial suppression). If a patient reported seeing two or three dots, the index was assigned a value of 2 (i.e., complete suppression).

### 2.8. Statistical analysis

Because our data were not normally distributed, we conducted a non-paired Wilcoxon rank-sum test to compare interocular suppression between IXT and control groups. In addition, we also conducted a paired Wilcoxon rank-sum test to compare near and far distance measurements within each participant group. The relationship between the suppression and clinical characteristics of IXT was also investigated in depth. For instance, we used Spearman rank correlation analysis to evaluate the relationship between interocular suppression measured in the butterfly test and clinical measurements of IXT, including the angle of deviation, Worth-4-Dot, stereopsis, and control scores. We performed the FDR (False Discovery Rate) approach to adjust the *p*-values for the multiple comparisons. Statistical analysis was performed using the SPSS 23.0 software package (SPSS Inc., Chicago, IL, United States). If *p*-values were less than 0.05 from a statistical test, we deemed the statistical result as significant.

## 3. Results

### 3.1. Reliability of our suppression test

**Figure 2:**
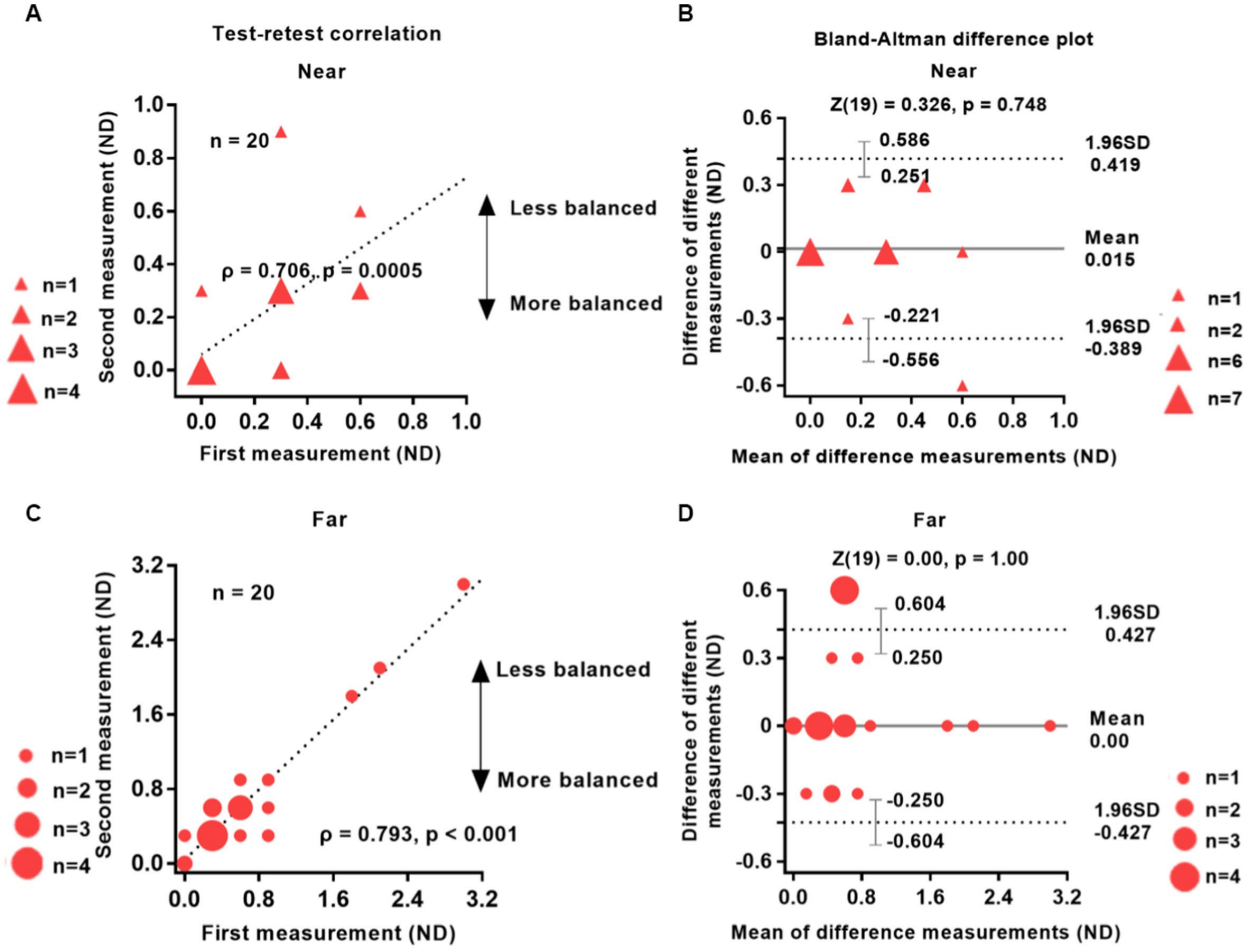
Reliability of the test–retest suppression test. The test–retest correlations for near and far distances (left column) and the Bland–Altman difference plot (right column) are shown. Twenty patients with IXT take part in the study. The data points vary in size, indicating different number of subjects, and the dashed line represents the identity line (slope = 1). The results of the two tests were significantly correlated: Near **(A)**, *ρ* = 0.706, *p* = 0.0005; Far **(C)**, *ρ* = 0.793, *p* < 0.001. The mean difference (bias) between the two measurements represented by the central black dashed line in panels **(B,D)** was 0.015 for near distance and 0.00 for far distance. The area enclosed by the dashed lines in panels **(B,D)** represents 95% confidence intervals, which denote the LoA (limits of agreement). The wider the LoA, the greater the variability of the data between the two test sessions of the visual test (Carkeet and Goh, 2018). Red solid triangle: IXT at near (*n* = 20); red solid circle: IXT at far (*n* = 20). ND, neutral density; SD, standard deviation.

Figure 2 shows the data obtained from the two independent test sessions of the interocular suppression test, involving 20 participants with IXT. Our analysis revealed a strong and significant correlation between the results of the first and second tests (near distance, *ρ* = 0.706; far distance, *ρ* = 0.793; *p* < 0.001, two-tailed Spearman rank correlation test). The Bland–Altman plots (Figures 2B,D) demonstrate minimal bias between the data collected from the two test sessions. Specifically, the bias was approximately 0.015 for near distance and 0.00 for far distance.

A two-tailed Wilcoxon rank sum test indicated no statistically significant difference between the two groups (*p* > 0.74). The 95% limits of agreement (LOA), defined as the mean difference ± 1.96 SD (Bland and Altman, 1999), were 0.419 and − 0.389 ND for near distance and 0.427 and − 0.427 ND for far distance, respectively. Additionally, the Wilcoxon rank sum test revealed no significant difference between the two tests: near distance (*Z* = 0.326, *p* = 0.748) and far distance (*Z* = 0.000, *p* = 1.000).

The eye position of patients with IXT was monitored using an eye tracker, which revealed a significant change in one patient and no significant change in the remaining 19 patients.

### 3.2. Greater interocular suppression in patients with intermittent exotropia than in age-matched control groups

**Figure 3:**
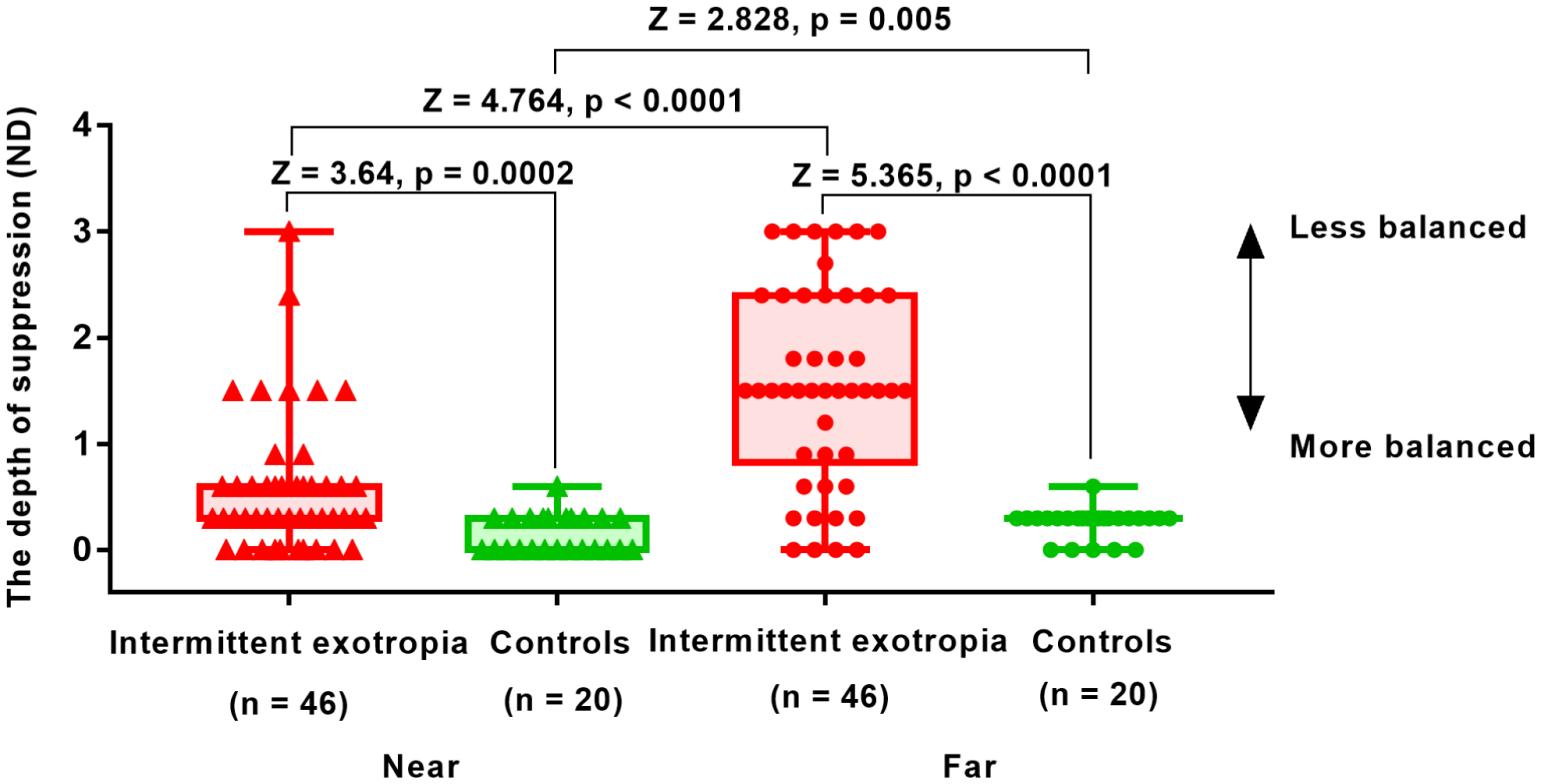
Interocular suppression in children with IXT and age-matched controls at near and far viewing distances. Patients (*n* = 46) and age-matched normal controls (*n* = 24) participated. The higher the value in the suppression test (*y*-axis), the greater the suppression. The suppression detected in patients with IXT was in the range of 0-3ND. The optical density of the filter was adjusted from 0.3ND (50% transmission) to 3ND (0.098% transmission) in 10 steps, with 0.3ND being 1 step. Red solid triangle: IXT at near (*n* = 46); red solid circle: IXT at far (*n* = 46); green solid triangle: controls at near (*n* = 24); green solid circle: controls at far (*n* = 24). ND, neutral density.

Figure 3 illustrates the results obtained from the suppression test conducted at near and far viewing distances in 46 patients with IXT. The difference in suppression between the far and near distances was found to be statistically significant (1.55 ± 0.93 vs. 0.57 ± 0.64; *Z* = 4.764, *p* < 0.0001). Controls had almost no suppression, with values of 0.15 ± 0.18 for near distance and 0.25 ± 0.14 for far distance. Comparatively, patients with IXT demonstrated greater suppression than the control group at both near distance (0.57 ± 0.64 vs. 0.15 ± 0.18; *Z* = 3.640, *p* = 0.0002) and far distance (1.55 ± 0.93 vs. 0.25 ± 0.14; *Z* = 5.365, *p* < 0.0001).

### 3.3. Correlation between interocular suppression and fusion, stereo function, fusion control, and deviation of intermittent exotropia

**Figure 4:**
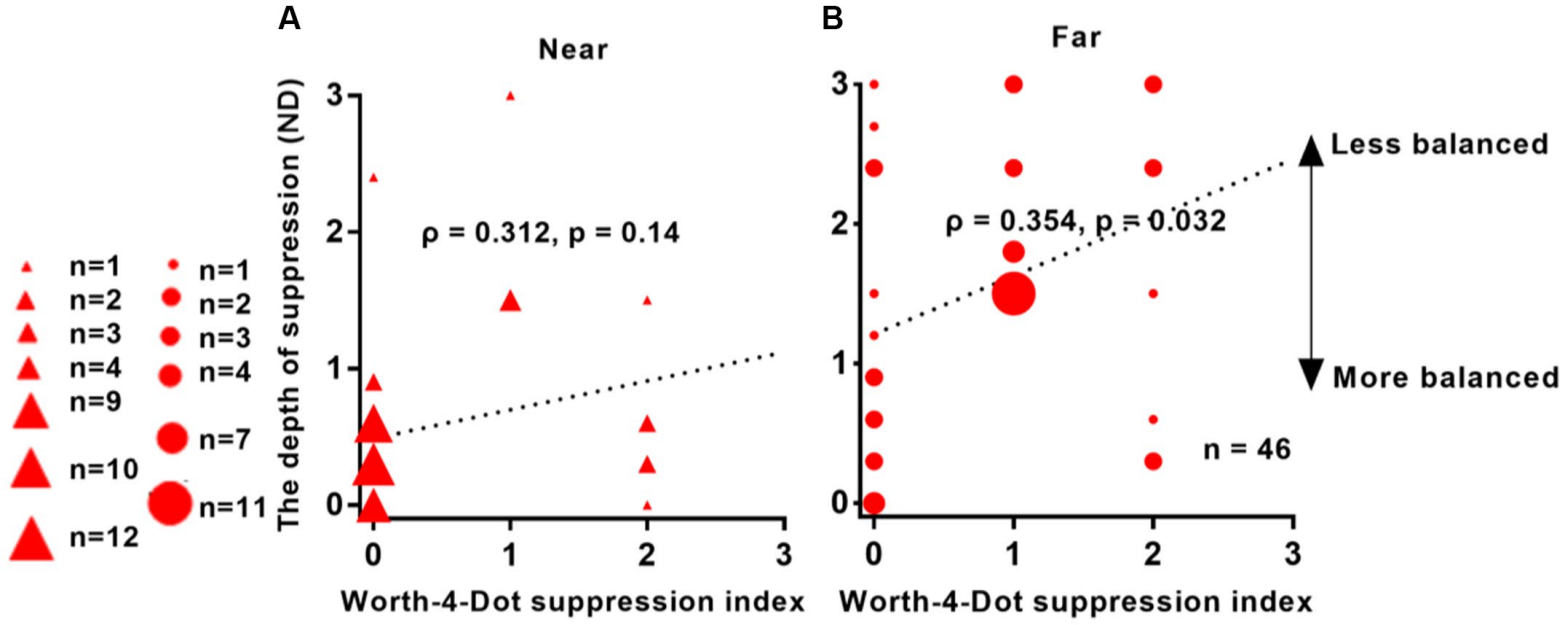
Relationship between interocular suppression and Worth-4-Dot test results in IXT patients. The *x*-axis represents the values obtained from the Worth-4-Dot test, where larger values indicate greater suppression. The *y*-axis represents the values obtained from the suppression test, where larger values also indicate greater suppression. The data points vary in size, indicating different number of subjects (46 patients participated). Statistical analysis was conducted using Spearman’s rank correlation, and the reported *p*-values were adjusted using FDR. Red solid triangle: IXT at near (*n* = 46); red solid circle: IXT at far (*n* = 46). **(A)** for near; **(B)** for far. ND, neutral density.

Figure 4 presents the results of a correlation analysis conducted between interocular suppression and the Worth-4-Dot test in 46 patients. The *p*-values were adjusted using the FDR approach. As depicted in Figure 4, a positive correlation was observed between interocular suppression and the Worth-4-Dot test results at the far viewing distance (*ρ* = 0.354, *p* = 0.032). However, no significant correlation was found for the near viewing distance (*ρ* = 0.312, *p* = 0.14).

**Figure 5:**
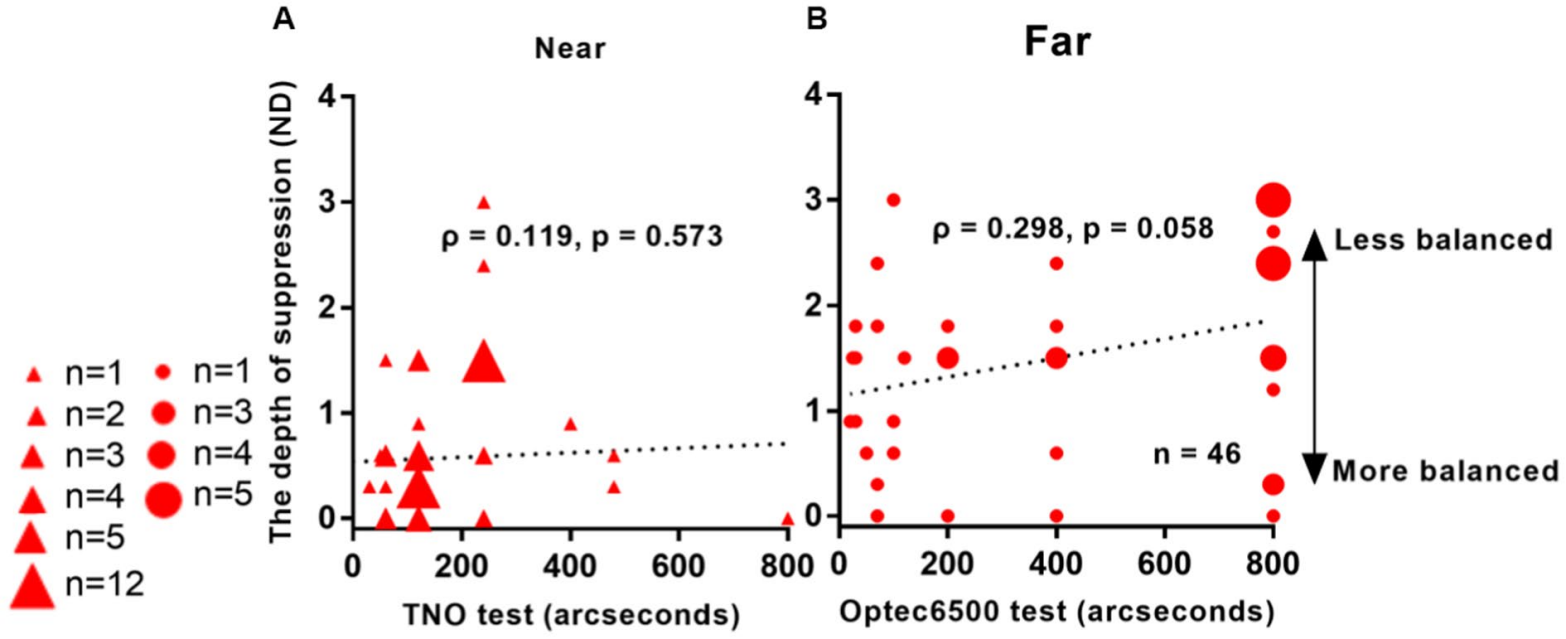
Relationship between interocular suppression and stereo acuity in IXT patients. The data points vary in size, indicating different number of subjects (46 patients participated). Statistical analysis for this comparison was conducted on the ranked data using Spearman’s rho. FDR adjusted p-values are reported here. Red solid triangle: IXT at near (*n* = 46); red solid circle: IXT at far (*n* = 46). **(A)** for near; **(B)** for far. ND, neutral density.

We conducted a correlation analysis between interocular suppression and stereoacuity from 46 Patients. FDR calibration was performed. Figure 5 demonstrates the results of this analysis. We observed an almost significant correlation between interocular suppression and stereoacuity, as obtained from the Optec 3500 test (*ρ* = 0.298, *p* = 0.058). However, no significant correlation was found between suppression depth and stereoacuity as assessed by the TNO test (*ρ* = 0.119, *p* = 0.573).

**Figure 6:**
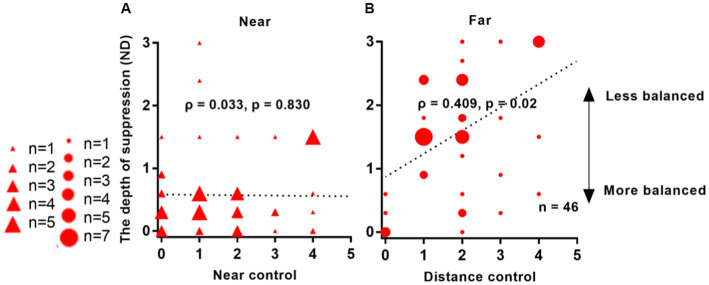
Relationship between interocular suppression and control scores in IXT patients. The data points vary in size, indicating different number of subjects (46 Patients participated). Statistical analysis was performed using Spearman’s Rho on rank data, and the reported p-values were adjusted using FDR. Red solid triangle: IXT at near (*n* = 46); red solid circle: IXT at far (*n* = 46). **(A)** for near; **(B)** for far. ND, neutral density.

Figure 6 demonstrates a significant correlation between interocular suppression and control scores measured at far distances from 46 patients: *ρ* = 0.409, *p* = 0.02. However, no correlation was found between suppression and control scores for near viewing distance: *ρ* = 0.033, *p* = 0.830.

**Figure 7:**
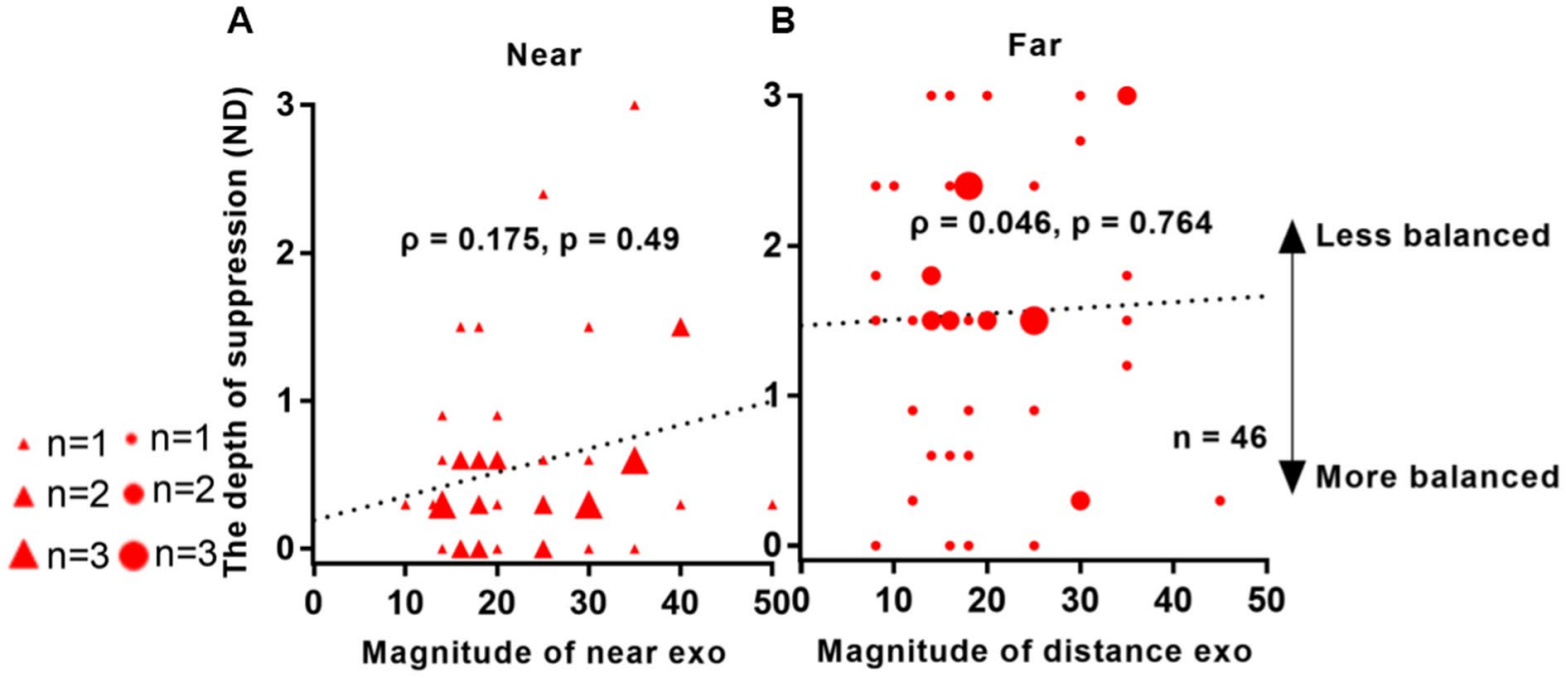
Relationship between interocular suppression and the angle of deviation in patients with IXT. The data points vary in size, representing different number of subjects (46 Patients participated). The statistical analysis employed Spearman rank correlation analysis, and the reported p-values were adjusted using FDR. Red solid triangle: IXT at near (*n* = 46); red solid circle: IXT at far (*n* = 46). **(A)** for near; **(B)** for far. ND, neutral density.

In Figure 7, no significant correlation was observed between suppression depth and angle of deviation in IXT patients. For near viewing distance, the correlation coefficient was *ρ* = 0.175, with a *p*-value of 0.49. Similarly, for far viewing distance, the correlation coefficient was *ρ* = 0.046, with a *p*-value of 0.764.

## 4. Discussion

Here, we introduced a new test that effectively measures interocular suppression and demonstrated its application in patients with IXT. Our test demonstrated good test–retest reliability, with significant changes in eye position observed in only one of the 20 IXT patients monitored and no changes in 19 patients. The concise nature of our test (2–5 min) and its ease of completion make it suitable for children. Previously, we have shown the efficacy of this method in capturing interocular suppression in amblyopia as well (Chen et al., 2019a,b, 2021).

The test–retest results presented in Figure 2 confirm the reliability of our test in IXT patients. Furthermore, we conducted a Spearman rank correlation test to examine the relationship between test–retest differences and the absolute value of the larger refraction in the patient’s eye among the 20 IXT patients who underwent the reliability test. The results indicated no correlation between the two variables (Near: *ρ* = 0.212, *p* = 0.370; Far: *ρ* = 0.011, *p* = 0.962), suggesting that the device response variation was unaffected by the patient’s refraction.

Consistent with previous research (Chen et al., 2019a), our study revealed significantly greater interocular suppression in patients with IXT compared to normal subjects. Additionally, we observed that interocular suppression was more pronounced at far distance than at near distance in IXT patients. Greater distance suppression was associated with poorer distance control and distance stereoscopic function. These findings align with previous studies that support the relationship between suppression and the ability to control exotropia (Etezad Razavi et al., 2012; Wakayama et al., 2013).

Our subjects underwent suppression testing while wearing frame glasses. It is possible that the use of contact lenses could impact the results. Exploring the potential differences between these two conditions could be an avenue for future investigation.

The correlation analysis between our suppression test and the Worth-4-Dot test revealed a positive correlation, although the correlation values were not high. This analysis aimed to compare our measurement with clinically available measures. However, clinically available measures can only capture a limited number of suppression levels, which can limit the results of the correlation analysis. Our strength lies in providing a more refined quantification of interocular suppression, offering ten levels for quantitative measurements. This result suggests a trend toward correlation, but not exact correspondence. On the one hand, the two measures differ in their level of refinement; on the other hand, the stimuli themselves are different, as our measurements use full-contrast stimuli rather than light as the visual target; furthermore, the results obtained by different methods of measuring suppression may not correspond exactly (Zhou et al., 2013a). We have the advantage of providing a simple, quantitative tool for measuring suppression that can be used by patients with IXT.

Ma et al.’s (2021) found that the fusion maintenance score was significantly correlated with distance and near control scores. They used a point light source. In contrast, we used black and white (i.e., full contrast) butterfly stimuli. Also, there are differences in environment brightness (52 lux & 15 lux) between their and our studies, so the results are different. PEDIG (Hatt et al., 2022) proposed an approach for detecting and assessing the severity of suppression in children with IXT, categorizing suppression on a 4-point scale, whereas our test employs a 10-point scale.

It is worth noting that interocular suppression can be influenced by environmental luminance (Zhou and Hess, 2016; Chen et al., 2021). Therefore, measurements of interocular suppression may yield different results under different luminance levels. In our study, interocular suppression was measured in a dimly lit room (15 lux), which may not reflect the same suppression levels in brighter lighting conditions. Standardizing the level of luminance should be emphasized when comparing suppression results across different studies, and future efforts should strive for agreement in illumination during evaluations.

Our results revealed that the worse the distance stereopsis and the worse the distance control, the greater the interocular suppression is. We believe that measuring interocular suppression can assist clinicians in predicting the progression of IXT and evaluating its control and stability. When making decisions about surgical intervention, the depth of suppression in patients should be taken into consideration, rather than solely focusing on the degree of deviation, especially in children who are still undergoing visual development. Additionally, Figueira and Hing (2006) reported that combining surgery with vision therapy yields higher success rates compared to surgery alone or vision/occlusion therapy.

According to current and previous studies (Figueira and Hing, 2006; Wakayama et al., 2013; Chen et al., 2019a), assessing interocular suppression in patients with IXT can provide valuable information for determining the optimal timing of surgical treatment and evaluating postoperative and functional outcomes. Our suppression test results demonstrate correlation with those obtained from standard clinical tests. Furthermore, our test offers a more precise measurement than the Worth-4-Dot test. The quantitative measurement of suppression provides advantages in monitoring subtle changes in the disease and may facilitate a more accurate quantification of disease severity. Additionally, in the future, combining our test with some other treatments such as binocular vision training may be helpful in treating binocular disorders (Chadnova et al., 2017; Sheynin et al., 2019; Baldwin et al., 2022). In short, our study suggests that our device could be a good choice to measure interocular suppression in young individuals with IXT.

## Data availability statement

The original contributions presented in the study are included in the article/supplementary material, further inquiries can be directed to the corresponding authors.

## Ethics statement

The studies involving human participants were reviewed and approved by the Ethical Committee of the Eye Hospital of Wenzhou Medical University. Written informed consent to participate in this study was provided by the participants’ legal guardian/next of kin.

## Author contributions

JQ, XY, JZ, JC, and HC: study design, securing funding, technical support, supervision, and critical revision of manuscript. HC and XJ: data acquisition. HC, WL, JZ, and QS: data analysis, interpretation, and statistical analysis. XY and JZ: manuscript drafting and critical revision. All authors read and approved the final manuscript.

## Funding

This study was supported by Health Commission of Zhejiang Province (Grant no. 2023KY154 to HC); National Natural Science Foundation of China (Grant no. 82070995 to XY); and Zhejiang Provincial Key Research and Development Program (Grant no. 2021C03102 to JQ). The sponsor or funding organization had no role in the design or conduct of this research.

## Conflict of interest

The authors declare that the research was conducted in the absence of any commercial or financial relationships that could be construed as a potential conflict of interest.

The authors JZ and XY declared that they were editorial board members of Frontiers, at the time of submission. This had no impact on the peer review process and the final decision.

## Publisher’s note

All claims expressed in this article are solely those of the authors and do not necessarily represent those of their affiliated organizations, or those of the publisher, the editors and the reviewers. Any product that may be evaluated in this article, or claim that may be made by its manufacturer, is not guaranteed or endorsed by the publisher.

## Footnotes

^1^ https://www.tobiipro.com/
